# 

**Published:** 1963-04

**Authors:** 


					?WW r'lwwtsi usJlh
.... or KiFDicirffi
At I li
April 1963
YY V
The
RECD. ! : !/?; i 9
*aJ u H JL IvO
Bristo
Medic
,4%m
incorporating the medical journal of the south west
Vol 78 (ii) NO 288

				

